# The Study of Cross-layer Optimization for Wireless Rechargeable Sensor Networks Implemented in Coal Mines

**DOI:** 10.3390/s16020171

**Published:** 2016-01-28

**Authors:** Xu Ding, Lei Shi, Jianghong Han, Jingting Lu

**Affiliations:** 1 Institute of Industry and Equipment Technology, Hefei University of Technology, Hefei 230009, China; dingxu@hfut.edu.cn (X.D.); jingtinglu_iiet@sina.com (J.L.); 2 School of Computer and Information, Hefei University of Technology, Hefei 230009, China; hanjh@hfut.edu.cn

**Keywords:** coal mines, wireless rechargeable sensor networks, wireless energy transfer, Lagrange dual problem, KKT conditions, cross-layer optimization

## Abstract

Wireless sensor networks deployed in coal mines could help companies provide workers working in coal mines with more qualified working conditions. With the underground information collected by sensor nodes at hand, the underground working conditions could be evaluated more precisely. However, sensor nodes may tend to malfunction due to their limited energy supply. In this paper, we study the cross-layer optimization problem for wireless rechargeable sensor networks implemented in coal mines, of which the energy could be replenished through the newly-brewed wireless energy transfer technique. The main results of this article are two-fold: firstly, we obtain the optimal relay nodes’ placement according to the minimum overall energy consumption criterion through the Lagrange dual problem and KKT conditions; secondly, the optimal strategies for recharging locomotives and wireless sensor networks are acquired by solving a cross-layer optimization problem. The cyclic nature of these strategies is also manifested through simulations in this paper.

## 1. Introduction

With the help of the rapid development of micro-electro-mechanical systems (MEMS), various types of sensors and actuators can be forged into an incredibly small size. Together with the state-of-the-art wireless communication technologies, wireless sensor networks (WSNs) are now playing an indispensable role in environment monitoring, target tracking, disaster rescuing and industrial process control [1].

Wireless sensor networks deployed in coal mines are assigned to gather environment temperature, humidity, seismic, gas leak information, *etc*. With this information at hand, we are able to evaluate the safety issues more precisely and provide workers with more reliable working conditions. However, due to the built-in nature of wireless sensor nodes, they are prone to different types of failures. Therefore, it is still not an easy task to ensure that these sensor nodes work properly over a long time. Since sensor nodes deployed in an underground environment may suffer from certain unattended ordeals, such as high temperature and humidity, the reasons accounting for failures of them may vary a great deal. Amongst all of these reasons, running out of energy supply takes up a large percentage of the reasons for node failure incidents.

Sensor nodes deployed in coal mines may be installed on the ceilings of mine tunnels or in walls; it is somehow difficult, if not impossible, to replace their batteries when their energy is depleted. Meanwhile, the energy harvesting techniques introduced by researchers may not be applied easily, since light, wind and ambient radio are all scanty commodities in an underground environment. Many other research works have been conducted in order to spend every bit of energy more wisely and efficiently to balance the communication load among all sensor nodes and to prolong the lifetime of WSNs. All of these works will be discussed in Section 2.

In this article, we discuss the corresponding issues of wireless rechargeable sensor networks that are implemented in coal mines with wireless energy transfer technique to prevent sensor nodes from energy depletion. As for wireless rechargeable sensor networks, this means that sensor nodes are equipped with certain wireless energy-receiving devices, such that they are able to receive energy charging remotely from a wireless power transferring source. The wireless energy transfer is not a brand-new concept. Early in the 20th century, this technique was advocated by a great inventor, Nikola Tesla. At the turn of the 21st century, the world witnessed the tremendous development of the inductive wireless power transfer technique. Kurs *et al.* from the Massachusetts Institute of Technology (MIT) proposed a new way to transfer energy remotely in a non-radiative way by means of the same magnetic resonance frequency among the transferor and the transferees. This technique is pretty suitable for wireless sensor networks in coal mines due to several reasons. Firstly, the way of wireless energy transfer is inductive, which will reduce the chance of fire accidents. Secondly, this type of wireless energy transfer nearly has no interference with this communication process.

The main contribution of this article is that we discuss the optimal cross-layer optimization problem for wireless sensor networks implemented for coal mines with the wireless energy transfer technique. The sensor nodes installed in coal mines are recharged remotely by mine locomotives passing through the tunnels with wireless energy transfer devices mounted on them. In order to make these sensor nodes immune to insufficient energy supply, the compatible working schemes for wireless sensor nodes and locomotives for recharging tasks are developed in this article. In order to obtain these schemes, two optimization problems are formulated and solved in this paper. The optimal working schemes are composed of the relaying nodes’ placement, the traveling paths taken by recharging locomotives, the charging time, *etc*.

The rest of this article is organized as follows: In Section 2, we introduce the wireless energy transfer technique, as well as some research results related to energy-aware issues in wireless sensor networks briefly. In Section 3, the working scenarios of wireless rechargeable sensor networks and recharging locomotives are introduced. In this section, we also introduce some preliminary knowledge and notations. In Section 4, the problem of optimal relay nodes’ placement is discussed. The problem is solved according to the Lagrange dual problem and the KKT (Karush–Kuhn–Tucker) conditions. In Section 5, the cross-layer optimization problem considering the working strategies of both sensor nodes and recharging locomotives is formulated and then reshaped into a linear programming problem with identical optimality. In Section 6, we perform simulations and analyze the numerical results. Section 7 concludes this paper.

## 2. Related Works

### 2.1. Wireless Energy Transfer

In the year 2007, Kurs from the Massachusetts Institution of Technology (MIT) published a breaking-through paper [2] in Science discussing transferring energy through magnetic fields between two coupled coils. The wireless energy transfer method introduced by this MIT research team has high efficiency and can be performed from a relatively long distance. Later on, this team co-founded the WiTricity Cooperation and manufactured several different types of wireless energy transfer devices, such as Prodigy, WiT-5000, WiT-3300, *etc*. Recently, many researchers enriched the theoretical results and practical usages of the newly-brewed wireless energy transfer technique. Zhong *et al.* found a way to make a three-coil wireless energy transfer system more efficient than a two-coil one [3]. Experiments were also conducted by Tang *et al.* to show the efficiency of wireless energy transfer between unsegmented and segmented coupling coils [4]. Li *et al.* wrote a paper introducing the potential usage of wireless energy transfer for electrical vehicles [5]. Wireless energy transfer may also be adopted to deliver energy to some in-body medical parts, such as heart pacemakers instead of complicated open chest surgery [6].

The wireless energy transfer method proposed by Kurs has several advantages compared to previous ones. First of all, this method adopts a non-radiative way to deliver energy between the transferor and transferees efficiently, even if there are ferrous obstacles amongst them. Secondly, the transfer range can be further increased by installing repeaters between the transferor and transferees. Moreover, this type of energy transfer has high directionality, *i.e.*, the energy delivery only succeeds among coils of the same magnetic resonance frequency. Besides, the energy transfer process has almost no interference with wireless communications, which makes it a suitable way to replenish the energy of sensor nodes. Khripkov *et al.* developed a data telemetry device, which can be charged remotely by a wireless energy transfer technique [7]. What is more, fortunately, the non-radiative nature of this wireless energy transfer technique will not emit electromagnetic waves and, hence, will not probably induce fire accidents or explosions, which is a huge advantage with respect to dealing with the safety-related issues in the complicated underground environment.

### 2.2. Energy-Related Issues in WSNs

Since sensor nodes in WSNs have a limited energy supply, they will sooner or later experience the depletion of their power sources. The whole network, therefore, may probably be paralyzed because of the failures of sensor nodes running out of energy. In dealing with this problem, many researchers devoted themselves to problems related to energy-aware issues in WSNs. Recently, many subtle energy-aware protocols have been proposed to use every drop of energy efficiently to prolong the lifetime of WSNs. Dervis *et al.* proposed a hierarchical clustering method and a corresponding data routing and cluster head election protocol to balance the remaining node energy, such that the lifetime of the wireless sensor network gets prolonged [8]. Selcuk *et al.* proposed an optimization problem to obtain the optimal energy-aware data routing scheme. The solution is acquired by implementing the differential evolution method [9]. Nicolas *et al.* studied the data routing recovery methods in wireless sensor networks while using controlled mobility to locate sensor nodes [10]. Zhao *et al.* made a comprehensive study on the medium access control (MAC) protocols applied in wireless sensor networks [11]. Jang *et al.* provided an energy-efficient MAC protocol to avoid overhearing and reduce contention and delay by asynchronous scheduling the wake-up time of neighboring nodes [12]. Cross-layer optimization methods were also well studied during these years in order to provide inter-layer solutions to energy-aware problems [13]. Indeed, these methods can squeeze out every bit of the node energy, balance the energy usage amongst different sensor nodes and prolong the lifetime of wireless sensor networks. Nevertheless, sensor nodes may still be inclined to malfunction due to their limited energy supply.

Many other researchers focus on developing suitable energy-harvesting technology to endow sensor nodes with a certain ability to harvest energy from the environment. The system architecture proposed by Yang *et al.* could acquire energy from solar power [14]. The sensor networks having a distributed fashion could achieve the optimal end-to-end network performance. Shigeta *et al.* developed a software control method for maximizing the sensing rate of WSNs, which could harvest energy from the ambient RF power [15]. Other researchers study the impact of energy-harvesting technology on the stability region of WSNs. Jeon and Ephremides characterized the stability region of the packet queues given energy-harvesting rates for the two-node slotted ALOHA system [16]. They also accurately assessed the effect of limited, but renewable, energy availability due to harvesting on the stability region by comparing against the case of having unlimited energy [17]. Pappas *et al.* provided inner and outer bounds on the stability region of a two-hop network with an energy harvesting source and relay [18]. They also discussed the relatively unexplored and important domain of energy harvesting in two-hop sensor networks by studying the maximum stable throughput region metric [19]. Krikidis *et al.* investigated the effects of network-layer cooperation in a wireless three-node network with energy-harvesting nodes and bursty data traffic. Additionally, they prove that orthogonal decode-and-forward cooperative schemes achieve a higher maximum stable throughput than a direct link for scenarios with poor energy arrival rates [20].

### 2.3. WSNs in Coal Mines

In recent years, safety-related issues and wireless sensor networks for coal mines are becoming more and more attractive [21]. Nazir *et al.* studied the routing scheme for emergency data for wireless sensor networking for coal mines [22]. Ruan *et al.* proposed an algorithm for task scheduling for underground wireless sensor networks based on distributed computing [23]. Menon *et al.* developed an early warning system (EWS) deployment strategy for wireless sensor networks in coal mines [24]. The corresponding power optimization strategies can switch sensor nodes to energy saving mode to reduce the energy consumption. However, research of wireless sensor networks implemented in coal mines with wireless energy transfer devices is still new and open to discussion.

## 3. The Working Scenario and Problems Statement

### 3.1. The Working Scenario

**Figure 1:**
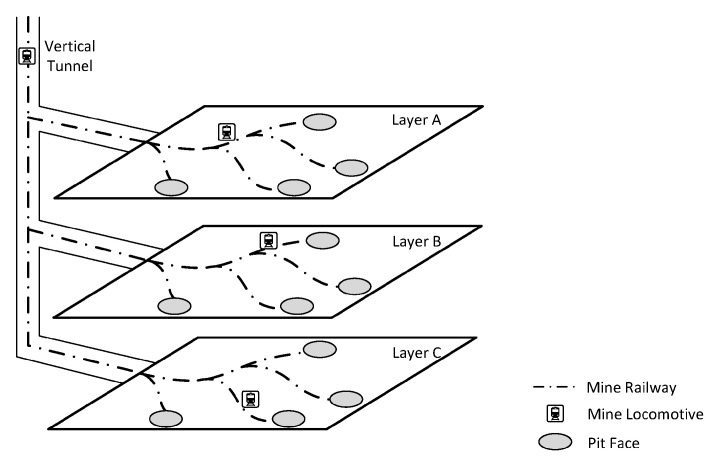
The sketch map of a coal mine.

The wireless sensor networks that we discuss here is intended to work in coal mines. A typical coal mine consists of several layers, as shown in Figure 1. The vertical well connects different layers together through mine tracks. The railroads paved on different layers lead the way to different pit faces where the mining operation proceeds. At certain locations of interest, different types of wireless sensor nodes are installed to gauge the humidity, temperature and gas density and to send these data back to a base station or a data center for future analysis. The data transmitting and receiving procedure will definitely consume a large portion of battery energy. Therefore, sooner or later, sensor nodes will encounter the problem of insufficient energy supply no matter what energy-saving techniques are adopted.

In order to make sensor nodes work properly without the problem caused by insufficient energy supply, recharging locomotives with wireless energy transfer devices mounted on them will cyclically stop by each sensor node and recharge it with a certain amount of energy, such that the energy of each sensor node will not fall below a certain level before the next round recharging task.

### 3.2. Problems Statement

The problems we discuss in this article can be split into two main sub-problems. Firstly, since each sensor node has a limited communication ability, relay nodes are a great need to keep the whole network connected. However, different criteria will lead to different sensor node placement strategies. In this paper, we study the optimal relay nodes’ placement with minimum overall energy usage as a criterion. The procedure of solving this problem is elaborated in Section 4. Secondly, as mentioned in previous sections, unmanned locomotives will charge sensor nodes to make sure that no sensor node will suffer from the shortage of energy supply. However, the design of charging strategies of locomotives is not trivial. Many factors, such as the traveling path, the charging power and charging durations for different sensor nodes, should be taken into consideration. In Section 5, in order to deal with this problem, we formulate an optimization problem and reshape it into a linear programming problem, which can be solved efficiently.

## 4. The Optimal Relay Nodes Placement with Respect to Minimum Overall Power Usage

### 4.1. Problem Formulation

Each sensor node has a limited communication ability; therefore, without pre-installed relay nodes, data acquired by sensor nodes may not be able to be piggybacked to the base station. In this section, we try to give the optimal scheme for relay nodes’ placement with respect to minimum overall power usage. Without losing the rigorous nature of mathematics, we resort to the Lagrange dual problem and KKT conditions in order to figure out the closed-form solution.

**Figure 2:**
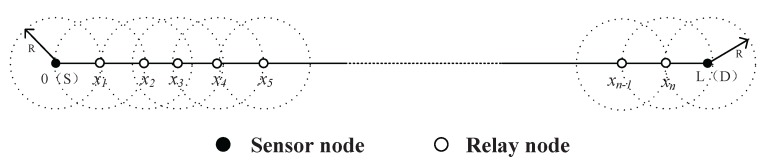
A sketch map for sensor nodes and relay nodes.

Before we step forward into the problem formulation, please allow us to introduce some notations used here. Since the networking issues among all layers are similar to each other, without any loss of generality, we take one layer of the mine for example. Since most coal mines are composed of tunnels, the topological structure of sensor networks of coal mines is somewhat linear. The optimization problem we formulated in this section is aimed to minimize the energy consumption of sensor and relay nodes along a linear structure. Assume that the distance between two sensor nodes is *L* meters. The communication range of each sensor node is *R* meters, and *R* < *L*. For the successful communication between these two sensor nodes, a certain amount of relay sensor nodes should be placed between them. As shown in Figure 2, there are *n* relay nodes placed successively at $x_{1},x_{2},\cdots ,x_{n}$, and each of these sensor nodes has a communication range of *R* meters. The positions of the two sensor nodes are denoted as $x_{0}$ and $x_{n+1}$. We assume that the relay nodes are only reliable for receiving and forwarding data in the following discussion.

The power usage of each node is composed of transmitting, receiving, processing and sensing data. Since the last two parts are relatively smaller compared to the first two factors and are independent of the optimization problem we discuss below, therefore, we omit the last two factors in the formulation of our optimization problem. The power usage of a node when transmitting data can be modeled as:

(1) $$\begin{array}{c} p_{T}^{i}\left(t\right)=\left(\Phi_{1}+\Phi_{2}d_{i}^{k}\right)r_{T}^{i}\left(t\right), \end{array}$$

where $p_{T}^{i}\left(t\right)$ is the transmitting power used by node *i* at time instance *t*, $d_{i}$ is the transmitting distance, *k* is often chosen between two and four and $r_{T}^{i}\left(t\right)$ is the transmitting data rate of node *i* at time instance *t*. The symbols $\Phi_{1}$ and $\Phi_{2}$ are power-related constants.

The receiving power used by node *i* can be modeled as:

(2) $$\begin{array}{c} p_{R}^{i}\left(t\right)=\rho r_{R}^{i}\left(t\right) \end{array}$$

where $p_{R}^{i}\left(t\right)$ is the power used for receiving data at time instance *t*, $r_{R}^{i}\left(t\right)$ is the receiving data rate and *ρ* is the power-related constant.

As shown in Figure 2, there are two sensor nodes and *n* relay nodes. By the definition of $x_{i}$ given in the previous section, we have the following equation,

(3) $$\begin{array}{c} \sum_{i=1}^{n+1}\left(x_{i}-x_{i-1}\right)=\sum_{i=0}^{n}d_{i}=L \end{array}$$

where $d_{i-1}=x_{i}-x_{i-1}$ is the distance between two successive sensor nodes.

Then, we formulate the following optimization problem, OPT-1:

$$\begin{array}{c} \begin{array}{ll} \text{min} & \int_{\tau }\left[\sum_{i=0}^{n}p_{T}^{i}\left(t\right)+\sum_{i=1}^{n+1}p_{R}^{i}\left(t\right)\right]dt\\ s.t. & \left(1)–(3\right)\\ & \left(0\leq d_{i}\leq R,i=0,\cdots ,n\right) \end{array} \end{array}$$

In this optimization problem, our objective is to minimize the energy used for transmitting data among sensor nodes and the corresponding relay nodes. The optimization variables are the number of relaying nodes n, which is an integer value, and the distance between two nodes $d_{i}$. The optimization constants are the power-related constants, *i.e.*, $\Phi_{1}$, $\Phi_{2}$ and *ρ*, the transmitting data rate of the first sensor node, $r_{T}^{0}\left(t\right)$.

Since all nodes should communicate properly, we have $0\leq d_{i}\leq R$. After solving this problem, we are able to obtain the optimal number and placement for relay nodes.

### 4.2. Solution to OPT-1

To solve this optimization problem, we might use the method of the Lagrange multiplier. However, the mathematical correctness and completeness of this method can only be ensured when finding extreme points for problems with equality constraints. The optimization problem formulated in Section 4.1 obviously has inequality constraints and an integer optimization variable. Therefore, the method of the Lagrange multiplier may not be applied easily without any criticism. In order to solve this problem, alternatively, we firstly divide OPT-1 into two sub-optimization problems. Secondly, we form the Lagrange dual problem of the first one and prove the strong duality of the prime and dual problems. Eventually, we combine these two optimization problems together to get the optimal number and placement for relay nodes.

By substituting the objective function with Equations (1) and (2), we have:

$$\begin{array}{c} \begin{array}{lll} \text{min} & \int_{\tau }\left[\sum_{i=0}^{n}\left(\Phi_{1}+\Phi_{2}d_{i}^{k}\right)r_{T}^{i}\left(t\right)+\sum_{i=1}^{n+1}\rho r_{R}^{i}\left(t\right)\right]dt & \\ s.t. & \left(3\right) & \\ & \left(0\leq d_{i}\leq R,i=0\cdots n\right) & \;. \end{array} \end{array}$$

The objective function can be further reshaped as:

(4) $$\begin{array}{c} \sum_{i=0}^{n}\left(\Phi_{1}+\Phi_{2}d_{i}^{k}\right)\int_{\tau }r_{T}^{i}\left(t\right)dt+\sum_{i=1}^{n+1}\rho \int_{\tau }r_{R}^{i}\left(t\right)dt=\sum_{i=0}^{n}\left(\Phi_{1}+\Phi_{2}d_{i}^{k}\right)f_{T}^{i}+\sum_{i=1}^{n+1}\rho f_{R}^{i} \end{array}$$

where $f_{T}^{i}=\int_{\tau }r_{T}^{i}\left(t\right)dt$ and $f_{R}^{i}=\int_{\tau }r_{R}^{i}\left(t\right)dt$ are the data that the i-th node transmits and receives during the time period *τ*. For the data integrity, during the time period *τ*, we have the following chain equation,

(5) $$\begin{array}{c} f_{T}^{0}=f_{R}^{1}=f_{T}^{1}=\cdots =f_{R}^{n}=f_{T}^{n}=f_{R}^{n+1}=f \end{array}$$

This chain equation indicates that during time period *τ*, the data transmitted by the i-th node are equal to the data received by it (in this article, we do not take into consideration the data aggregation and data compression at each node).

After expanding the objective function, we have:

$$\begin{array}{c} \begin{array}{ll} \text{min} & \left(n+1\right)\left(\Phi_{1}+\rho \right)f+\Phi_{2}f\sum_{i=0}^{n}d_{i}^{k}\\ s.t. & \left(3\right)\\ & \left(0\leq d_{i}\leq R,i=0\cdots n\right) \end{array} \end{array}$$

The Constraints (1) and (2) are omitted here in that they have been already plugged into the objective function. The optimization variables are the number of relay nodes *n* and the distance between two successive nodes $d_{i}$. To solve this optimization problem, we firstly divide it into two sub-optimization problems.

For a given number *n*, we can firstly solve the following optimization problem OPT-2,

$$\begin{array}{c} \begin{array}{lll} \text{min} & \Phi_{2}f\sum_{i=0}^{n}d_{i}^{k} & \\ s.t. & \sum_{i=0}^{n}d_{i}-L=0 & \\ & d_{i}-R\leq 0 & \left(i=0\cdots n\right)\\ & -d_{i}\leq 0 & \left(i=0\cdots n\right) \end{array} \end{array}$$

It is not difficult to verify that this optimization problem is a convex problem since the objective function and all of the constraints are convex.

Then, the Lagrangian of this prime optimization problem is:

(6) $$\begin{array}{c} L\left(d_{i},\lambda_{1i},\lambda_{2i},v\right)=\Phi_{2}f\sum_{i=0}^{n}d_{i}^{k}+\sum_{i=0}^{n}\lambda_{1i}\left(-d_{i}\right)+\sum_{i=0}^{n}\lambda_{2i}\left(d_{i}-R\right)+v\left(\sum_{i=0}^{n}d_{i}-L\right) \end{array}$$

The Lagrangian dual function is defined as:

(7) $$\begin{array}{c} G\left(\lambda_{1i},\lambda_{2i},v\right)=\underset{d_{i}}{\text{inf}}\left\{L\left(d_{i},\lambda_{1i},\lambda_{2i},v\right)\right\}=\underset{d_{i}}{\text{inf}}\left\{\Phi_{2}f\sum_{i=0}^{n}\lambda_{1i}\left(-d_{i}\right)+\sum_{i=0}^{n}\lambda_{2i}\left(d_{i}-R\right)+v\left(\sum_{i=0}^{n}d_{i}-L\right)\right\} \end{array}$$

where the $\underset{d_{i}}{\text{inf}}$ stands for the infimum over all $d_{i}$.

The Lagrange dual problem of the prime problem is:

$$\begin{array}{c} \begin{array}{ll} \text{max} & G(\lambda_{1i},\lambda_{2i},v)\\ s.t. & \lambda_{1i},\lambda_{2i}\geq 0 \end{array} \end{array}$$

In general cases, the optimal value of the dual problem always provides a loose lower bound of the optimal value of the prime one. However, in this case, we can prove that the strong duality holds for the prime and dual problems, that is the optimal values of the prime and dual problem are identical.

**Theorem** **1.**  The strong duality of the prime optimization problem OPT-2 and its Lagrange dual problem holds, i.e., the optimal values of these two problem are identical.

The proof of this theorem is not trivial, and we leave it to the Appendix section for a better intelligibility. The next theorem will show a necessary condition for achieving the optimality of OPT-1.

**Theorem** **2.**  The optimality of OPT-1 can be achieved only if relay nodes are placed at the equal partition points between two sensor nodes.

**Proof.**  Since the prime optimization problem OPT-2 is a convex problem and the strong duality holds, we can use the KKT conditions to figure out the optimal value of both the prime and dual optimization problems.

The KKT conditions for OPT-2 for a given n are listed below:

(8) $$\begin{array}{c} \left\{\begin{array}{l} \sum_{i=0}^{n}d_{i}-L=0\\ d_{i}-R\leq 0\\ -d_{i}\leq 0\\ \lambda_{1i}\left(-d_{i}\right)=0\\ \lambda_{2i}\left(d_{i}-R\right)=0\\ \lambda_{1i}\geq 0\\ \lambda_{2i}\geq 0\\ \frac{\partial (\Phi_{2}f\sum_{i=0}^{n}d_{i}^{k}+\sum_{i=0}^{n}\lambda_{1i}\left(-d_{i}\right)+\sum_{i=0}^{n}\lambda_{2i}\left(d_{i}-R\right)+v\left(\sum_{i=0}^{n}d_{i}-L\right))}{\partial d_{i}}=0 \end{array}\right. \end{array}$$

After solving this set of equations, we can draw the conclusion that,

(9) $$\begin{array}{c} d_{0}=d_{1}=\cdots =d_{n}=\frac{L}{n+1} \end{array}$$

which means that relay nodes must be put at the equal partition points between two sensor nodes to get the minimum overall energy consumption, since the first term of the objective function of OPT-1 is constant given the value of *n*.

The next step is to decide how many relay nodes should be installed. Since relay nodes should be placed at the equal partition points, the optimization problem OPT-1 can be rewritten as the following optimization problem, denoted as OPT-3,

$$\begin{array}{c} \begin{array}{ll} \text{min} & \left(n+1\right)\left(\Phi_{1}+\rho \right)f+\Phi_{2}f\frac{L^{k}}{{\left(n+1\right)}^{k-1}}\\ s.t. & n\;\text{is}\;\text{an}\;\text{integer} \end{array} \end{array}$$

The variable *n* of this optimization problem must have an integer value. However, we can first take the unconstrained problem into consideration, *i.e.*,

$$\begin{array}{c} \text{min}\;\left(n_{c}+1\right)\left(\Phi_{1}+\rho \right)f+\Phi_{2}f\frac{L^{k}}{{\left(n_{c}+1\right)}^{k-1}} \end{array}$$

By calculating the second derivative of the objective function, we could find out that this function is strict convex when $n_{c}$ is greater than zero. Therefore, this problem can be solved by calculating the stationary points of the objective function, *i.e.*, if any stationary point exists when *n* is greater than zero, it also must be the minimum point. In order to calculate the stationary point, we first calculate the first derivative of the objective function. We have:

(10) $$\begin{array}{c} \begin{array}{ll} & \partial \left(\left(n_{c}+1\right)\left(\Phi_{1}+\rho \right)f+\Phi_{2}f\frac{L^{k}}{{\left(n_{c}+1\right)}^{k-1}}\right)/\partial n_{c}\\ = & \left(\Phi_{1}+\rho \right)f-\left(k-1\right)\Phi_{2}f\frac{L^{k}}{{\left(n_{c}+1\right)}^{k}} \end{array} \end{array}$$

Therefore,

(11) $$\begin{array}{c} n_{c}=L{\left(\frac{\left(k-1\right)\Phi_{2}}{\Phi_{1}+\rho }\right)}^{1/k}-1 \end{array}$$

From the property of convex functions, we have the following conclusions:

- If $L{\left(\frac{\left(k-1\right)\Phi_{2}}{\Phi_{1}+\rho }\right)}^{1/k}-1$ is an integer and $L{\left(\frac{\left(k-1\right)\Phi_{2}}{\Phi_{1}+\rho }\right)}^{1/k}-1\geq \left\lfloor \frac{L}{R}\right\rfloor$, then $n=L{\left(\frac{\left(k-1\right)\Phi_{2}}{\Phi_{1}+\rho }\right)}^{1/k}-1$;
- If $L{\left(\frac{\left(k-1\right)\Phi_{2}}{\Phi_{1}+\rho }\right)}^{1/k}-1>\left\lfloor \frac{L}{R}\right\rfloor$, but $L{\left(\frac{\left(k-1\right)\Phi_{2}}{\Phi_{1}+\rho }\right)}^{1/k}-1$ is not endowed with an integer value, then the value of *n* is chosen from $\left\lfloor L{\left(\frac{\left(k-1\right)\Phi_{2}}{\Phi_{1}+\rho }\right)}^{1/k}-1\right\rfloor$ and $\left\lceil L{\left(\frac{\left(k-1\right)\Phi_{2}}{\Phi_{1}+\rho }\right)}^{1/k}-1\right\rceil$;
- If $L{\left(\frac{\left(k-1\right)\Phi_{2}}{\Phi_{1}+\rho }\right)}^{1/k}-1<\left\lfloor \frac{L}{R}\right\rfloor$, then the value of *n* is $\left\lfloor \frac{L}{R}\right\rfloor$.

Next, the correctness of solving OPT-1 via the given procedure can be validated by contradiction.

Assume that the value *n* and the relay nodes’ placement is not optimal, *i.e.*, there exist another value $n^{\prime }$ and a relay nodes’ placement method that lead to a smaller objective value of OPT-1. However, for a given $n^{\prime }$, due to the result of OPT-2, the objective function can only be minimized by placing relay nodes at the equal partition points. Then, OPT-1 is transformed into OPT-3. Since the value of n is obtained by solving OPT-3, the objective function value must be smaller than that acquired by $n^{\prime }$, which causes the contradiction. Therefore, the optimality of OPT-1 can be ensured when following the given solution procedure. ☐

## 5. The Optimal Working Strategies for Wireless Rechargeable Sensor Networks

In the last section, we discuss the relay nodes’ placement issue. After that, we will study the working strategies for sensor nodes and the recharging locomotives. The working strategies consist of the data routing protocols, roaming path, energy charging time, *etc.*. In order to obtain these strategies, we formulated another optimization problem and reshape it into a linear programming problem, which can be solved efficiently.

### 5.1. Problem Formulation

Assume that there are *N* nodes, including sensor nodes and relay nodes, deployed in one layer of the coal mine. Each node is equipped with a wireless rechargeable battery. The initial energy of each node is denoted as $E_{max}$. The minimum energy level to maintain the routine duty of sensor nodes is denoted as $E_{min}$, where $E_{min}=\alpha E_{max}\;\left(0\leq \alpha \leq 1\right)$.

Before formulating the optimization problem, we should first introduce some constraints that nodes and recharging locomotives should comply with. First of all, all nodes, including sensor nodes and relay nodes, should satisfy the following equation:

(12) $$\begin{array}{c} r^{i}+r_{R}^{i}\left(t\right)=r_{T}^{i}\left(t\right) \end{array}$$

where $r^{i}$ is the data rate generated by nodes *i*. For relay nodes, $r^{i}$ is zero. $r_{R}^{i}\left(t\right)$ is the data rate received by node *i* at time instance *t*. $r_{T}^{i}\left(t\right)$ is the data rate transmitted by node *i* at time instance *t*. In other words, Equation (12) is the network flow constraint.

The second constraint is the power usage constraint. Each sensor node should satisfy:

(13) $$\begin{array}{c} p^{i}\left(t\right)=p_{R}^{i}\left(t\right)+p_{T}^{i}\left(t\right)=\rho r_{R}^{i}\left(t\right)+\left(\Phi_{1}+\Phi_{2}d_{i}^{k}\right)r_{T}^{i}\left(t\right) \end{array}$$

We want to design cyclic work strategies for the recharging locomotive, that is it will roam periodically along the traveling path and charge each sensor node with a certain amount of energy. The period of each workload of recharging locomotives is denoted as *τ*. In order to meet the periodic requirement, the energy consumed during each period should be the same as that recharged, *i.e.*,

(14) $$\begin{array}{c} \int_{\tau }p_{i}\left(t\right)dt=U\tau_{i} \end{array}$$

where *U* is the power used for recharging nodes and *τ* is the length of a single charging period.

During each period, in order to keep sensor nodes working properly, the energy level of each node should not fall below $E_{min}$ and should not exceed $E_{max}$, which is the upper bound of battery energy. Therefore, we have the following constraint:

(15) $$\begin{array}{c} E_{min}\leq E_{i}\left(t\right)\leq E_{max} \end{array}$$

There are also some constraints that the wireless energy transfer device should satisfy. First of all, since the wireless energy transfer device should stop by each node, we denote the visiting order of nodes as $P=\{\pi_{0},\pi_{1},\cdots ,\pi_{0}\}$, where $\pi_{0}$ stands for the sojourn spot at which the locomotive receives services, such as replacing the energy source, checking mechanical structure status, and so on. $\pi_{i}$ stands for the i-th node visited by the wireless energy transfer device. The length of the traveling path taken by the wireless energy transfer device is denote as $D_{P}$, and the time spent on roaming is denoted as $\tau_{P}$. Then, we have,

(16) $$\begin{array}{c} \tau_{p}=D_{P}/V \end{array}$$

where *V* is the moving velocity of the recharging locomotive.

*τ* could be further divided into three parts, that is,

(17) $$\begin{array}{c} \tau =\sum_{i}\tau_{i}+\tau_{P}+\tau_{s} \end{array}$$

where $\tau_{s}$ is the time spent on staying at the sojourn spot and $\tau_{i}$ is the time spent on charging the i-th node along the traveling path.

In order to minimize the interference introduced by locomotives mounted with wireless energy devices when roaming around the sensor networks, we hope that locomotives could stay at sojourn spots as much as possible while all of the Constraints (12)–(17) will be satisfied. Therefore, we have the following optimization problem, OPT-4,

$$\begin{array}{c} \begin{array}{ll} \text{max} & \tau_{s}/\tau \\ s.t. & \left(12)–(17\right) \end{array} \end{array}$$

The objective function stands for our pursuit of more time spent at sojourn spots in each charging period. The optimization variables of OPT-4 are the traveling path *P*,$\tau_{s}$,$\tau_{i}$,*τ*,$r_{R}^{i}\left(t\right)$ and $r_{T}^{i}\left(t\right)$, and the rest of the symbols stand for the optimization constants.

### 5.2. The Simplification and Linearization of OPT-4

The optimization problem formulated in the last subsection is not easy to solve for several reasons. First of all, the optimization problem has variables that are continuous over time. Secondly, the constraints of this problem contain integration terms and proportional terms, which make this problem nonlinear. Therefore, it is not an easy job to find an efficient algorithm to obtain the optimal solution. Instead of providing a heuristic algorithm, in this paper, we try to reach the optimal solution of OPT-4 by simplifying and linearizing it.

First of all, we want to introduce two properties that are useful when transforming the original problem into a linear programming problem.

**Property** **1.**  The energy of each node reaches its peak when the locomotive mounted with the wireless transfer device finishes recharging it. Moreover, its energy falls to the bottom when the locomotive arrives at this node.

**Property** **2.**  If the batteries of nodes are fully recharged, that is the energy of each battery reached when the locomotive finishes recharging task, the optimal value of OPT-4 remains the same.

With these two properties, we are able to slightly change the form of OPT-4. For the constraint (15), we can rewrite it as:

(18) $$\begin{array}{c} E_{i}\left(t_{i}\right)\geq E_{min},E_{i}\left(t_{i}+\tau_{i}\right)=E_{max} \end{array}$$

where $t_{i}$ denotes the arriving time of the recharging locomotive at the i-th node. Then, we have the new optimization problem, OPT-5, with identical optimality.

$$\begin{array}{c} \begin{array}{ll} \text{max} & \tau_{s}/\tau \\ s.t. & \left(12)–(18\right) \end{array} \end{array}$$

We will then try to take the integration terms out to simplify OPT-5. The integration terms are introduced by Constraint (14). We first substitute Equation (14) with Equations (12) and (13), then we have:

(19) $$\begin{array}{c} \rho \int_{\tau }r_{R}^{i}\left(t\right)dt+\left(\Phi_{1}+\Phi_{2}d_{i}^{k}\right)\int_{\tau }r_{T}^{i}\left(t\right)dt=U\tau_{i} \end{array}$$

According to the Lagrange mean value theorem, there exists $r_{R}^{i}$ and $r_{T}^{i}$, which satisfy:

(20) $$\begin{array}{c} \rho r_{R}^{i}\tau +\left(\Phi_{1}+\Phi_{2}d_{i}^{k}\right)r_{T}^{i}\tau =U\tau_{i} \end{array}$$

For Equation (12), we have,

(21) $$\begin{array}{c} r^{i}+r_{R}^{i}=r_{T}^{i} \end{array}$$

Then, we have the optimization problem OPT-6,

$$\begin{array}{c} \begin{array}{ll} \text{max} & \tau_{s}/\tau \\ s.t. & \left(16)–(21\right) \end{array} \end{array}$$

The constraint (14) is omitted since we plug it into Equation (20).

In OPT-6, we use $r_{R}^{i}$ and $r_{T}^{i}$ instead of continuous variables. The next theorem will show the equal optimality of OPT-5 and OPT-6.

**Theorem** **3.**  *If we use discrete variables* $r_{R}^{i}$ *and* $r_{T}^{i}$ *instead of* $r_{R}^{i}\left(t\right)$ *and* $r_{T}^{i}\left(t\right)$, *the optimal values of both OPT-5 and OPT-6 are the same, that is OPT-5 and OPT-6 have equal optimality*.

**Proof.**  The proof of this theorem can be divided into two steps. On the one hand, since we use $r_{R}^{i}$ and $r_{T}^{i}$ instead of $r_{R}^{i}\left(t\right)$ and $r_{T}^{i}\left(t\right)$, the flexibility of variables is of course compromised to a certain extent. The optimal values of OPT-5 and OPT-6 are denoted as $O_{5}$ and $O_{6}$. Then, we have $O_{5}\geq O_{6}$. On the other hand, for each feasible solution of OPT-5, we are always able to construct a corresponding solution to OPT-6 that yields the same objective value, which means $O_{5}\leq O_{6}$. Therefore, we have $O_{5}=O_{6}$. ☐

The next theorem will tell us the optimal traveling path that the locomotive should take to achieve the optimality.

**Theorem** **4.**  The optimal traveling path that the recharging locomotive should take is the shortest roaming path connecting all nodes and the sojourn spot.

**Proof.**  The proof of this theorem is based on contradiction, that is if a solution to the optimization problem yields the optimal value while the locomotive does not follow the shortest roaming path, we can construct a new solution from it that yields a better objective value while the locomotive travels along the shortest roaming path. Intuitively, if the locomotive spends less time on traveling, it will be rewarded more time staying at the sojourn spot. ☐

For a given shortest roaming path in a coal mine and a given moving velocity, the constraint (15) can also be omitted in OPT-6. Then, we have the following optimization problem, OPT-7:

$$\begin{array}{c} \begin{array}{ll} \text{max} & \tau_{s}/\tau \\ s.t. & \left(17)–(21\right) \end{array} \end{array}$$

By exploiting the cyclic nature, OPT-7 can be further reshaped as:

$$\begin{array}{c} \begin{array}{ll} \text{max} & \tau_{s}/\tau \\ s.t. & r^{i}+r_{R}^{i}=r_{T}^{i}\\ & U\tau_{i}-\left(\rho r_{R}^{i}+\left(\Phi_{1}+\Phi_{2}d_{i}^{k}\right)r_{T}^{i}\right)\tau_{i}\leq E_{max}-E_{min}\\ & \tau =\sum_{i}\tau_{i}+\tau_{P}+\tau_{S} \end{array} \end{array}$$

We denote this problem as OPT-8, and it can be further linearized as:

$$\begin{array}{c} \begin{array}{ll} \text{max} & \xi_{S}\\ s.t. & r^{i}+r_{R}^{i}=r_{T}^{i}\\ & \xi_{0}-\xi_{S}-\left(\rho r_{R}^{i}+\left(\Phi_{1}+\Phi_{2}d_{i}^{k}\right)r_{T}^{i}\right)\left(1-\xi_{i}\right)\frac{\tau_{P}}{E_{max}-E_{min}}\geq 0\\ & \sum_{k=0}^{N}\xi_{k}=1 \end{array} \end{array}$$

The above optimization problem is OPT-9, and the reshaping process is elaborated in the Appendix section. Now, OPT-9 is a linear programming problem with optimization variables $\xi_{0}$, $\xi_{S}$, $r_{R}^{i}$ and $r_{T}^{i}$. Additionally, this linear programming program can be solved efficiently by some commercial tools, such as LINDO API and CPLEX.

## 6. Simulations and Numerical Analysis

### 6.1. Simulation Scenario

**Figure 3:**
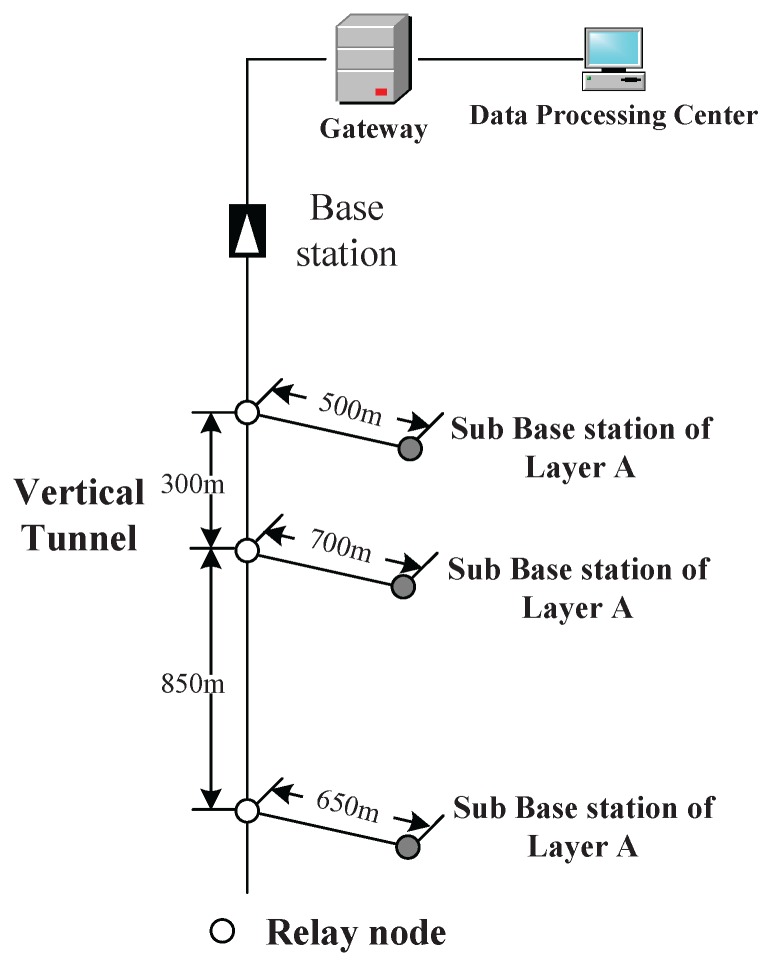
A sketch map for a coal mine with three layers.

**Figure 4:**
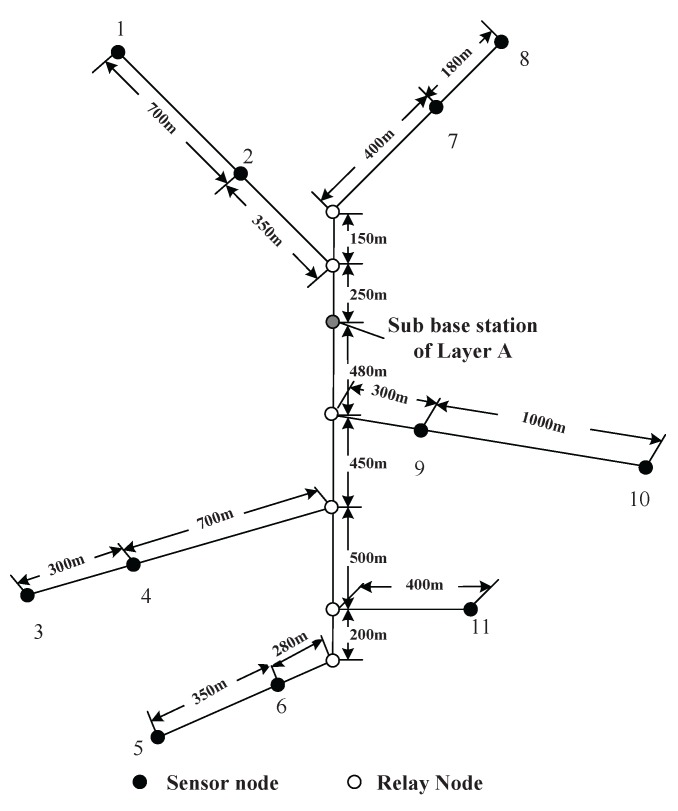
A sketch map for the sensor nodes deployment in Layer A.

The simulation scenario is a coal mine with three layers (Layers A, B and C), as shown in Figure 3. The vertical tunnel connects the three layers. In this section, the simulation will be conducted at the vertical tunnel and one horizontal layer (Layer A). The figure of Layer A is shown in Figure 4. At each intersection of the vertical and horizontal tunnels, there is one sub-base station, which has the duty of collecting the data of its own layer and forwarding them to the base station.

Several sensor nodes are installed along the mine tracks of the vertical tunnel and horizontal tunnels. There are also some of them installed at pit faces to collect the environmental information. At the entrance of each branch of horizontal tunnel, a relay node is placed in case of wireless signal loss. The nodes’ placement is shown in Figure 4. The sensor nodes are labeled with dot symbols. The relay nodes at branch entrances and the sub-base station are denoted as hollow circles. The sojourn spot of the recharging locomotive is located at the sub-base station. After recharging each node, the recharging locomotive will stay at the sojourn spot receiving maintenance.

### 6.2. Simulation Parameters

The rate of data generated by each sensor nodes is listed in Table 1. The data rates are random numbers ranging from 10–20 kb/s. The lengths between each node are labeled in Figure 4. The moving velocity of the recharging locomotive is 5 m/s. The energy-related parameters are listed in Table 2. The communication range of each node is 100 m.

**Table 1:** The *r_i_* of each sensor node in Layer A.

Node No.	*r_i_* (kb/s)	Node No.	*r_i_* (kb/s)	Node No.	*r_i_* (kb/s)
1	17	5	14	9	19
2	16	6	18	10	19
3	19	7	15	11	10
4	13	8	18		

**Table 2:** Values of parameters related to power usage.

Notation	Value	Notation	Value	Notation	Value
*E_max_*	10.8 kJ	*U*	5 W	Φ_1_	50 nJ/b
*E_min_*	540 J	*ρ*	50 nJ/b	Φ_2_	0.013 pJ/(b m^4^)
*v*	5 m/s				

### 6.3. Simulation Tools

The simulation codes are programmed and executed on a ThinkPad W530. The CPU of this computer is an Intel Core i7-3840, and its RAM is 16 GB. The operation system is Win7 Home Premium. We use Microsoft VS 2010 and the LINDO API to solve the optimization problem. MATLAB R2010a is used to analyze the data and draw the corresponding pictures.

### 6.4. Simulation Results

In the simulation, we compare the charging strategy corresponding to the minimum energy consumption routing with the one corresponding to the minimum hop routing. For minimum hops and minimum power usage routing schemes, the relay nodes’ placement for Layer A is listed in Table 3. The optimal numbers and deploying locations of relay nodes in minimum energy consumption mode are calculated according to OPT-1, OPT-2 and OPT-3.

**Table 3:** Relay nodes’ placement for both minimum hops and minimum overall energy consumption routings in Layer A. BE, branch entrance.

Placement	No. of Relay Nodes for Minimum Hop Routing	No. of Relay Nodes for Minimum Overall Power Usage Routing (Theoretically)	No. of Relay Nodes for Minimum Overall Power Usage Routing (Floor Number)	No. of Relay Nodes for Minimum Overall Power Usage Routing (Ceiling Number)
1-2	6	8.8	8	9
2-BE	3	3.9	3	4
3-4	2	3.2	3	4
4-BE	6	8.8	8	9
5-6	3	3.9	3	4
6-BE	2	2.9	2	3
7-8	1	1.5	1	2
7-BE	3	4.6	4	5
9-10	9	13	13	14
9-BE	2	3.2	3	4
11-BE	3	4.6	4	5

**Figure 5:**
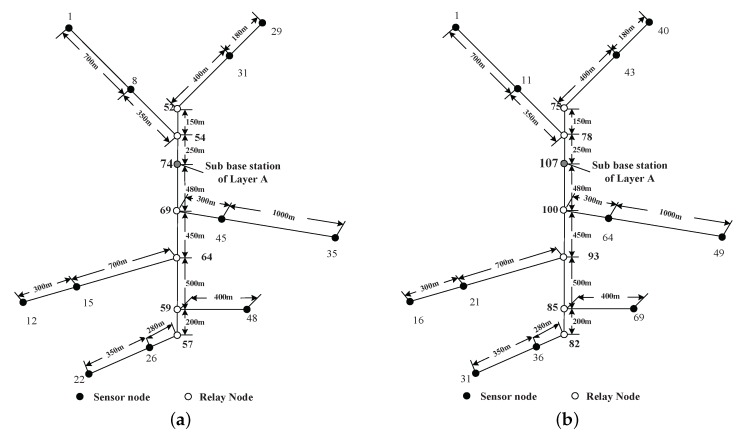
The node numbers after adding relay nodes for both routing schemes in Layer A. (**a**) Minimum hop routing. (**b**) Minimum overall power usage routing.

The notation “1-2” in the first column means relay nodes should be placed on the equal partition points between Sensor Nodes 1 and 2. The notation “2-BE” means relay nodes should be placed on the equal partition points between Sensor Node 2 and the node places at the entrance of this branch (BE). The second column tells us how many relay nodes should be placed according to the minimum hop routing. The third column shows the optimal number of nodes theoretically according to the minimum overall power usage routing. Columns 3 and 4 show the floor and ceiling numbers. The locations and numbers of nodes are shown in Figure 5.

**Figure 6:**
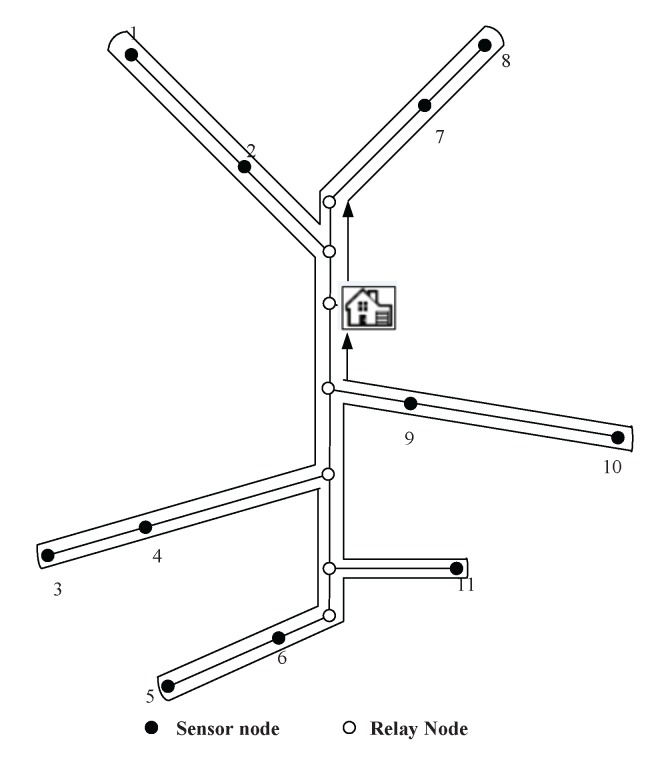
The optimal traveling path of the recharging locomotive in Layer A.

The optimal traveling path of the recharging locomotive is shown in Figure 6. After solving OPT-9, we have the optimal working strategies for both routing minimum energy consumption and minimum hop schemes, which are manifested in Table 4 and Table 5.

**Table 4:** The working strategies for minimum hop routing in Layer A.

Node No.	Arrival Time (s)	Recharging Duration (s)	Remaining Battery Energy (J)	Node No.	Arrival Time (s)	Recharging Duration (s)	Remaining Battery Energy (J)
56	437, 442	267	9462	16	454, 418	382	8890
55	437, 727	342	9091	15	454, 820	382	8890
54	438, 085	342	9091	14	455, 223	382	8890
53	438, 443	342	9091	13	455, 625	382	8890
52	438, 800	342	9091	12	456, 027	382	8890
34	439, 162	342	9091	63	456, 429	382	8890
33	439, 524	342	9091	62	457, 032	382	8890
32	439, 886	438	8608	61	457, 434	382	8890
31	440, 345	508	8259	60	457, 836	681	7397
30	440,871	508	8259	59	458,538	764	6983
29	441,398	508	8259	58	459,322	764	6983
11	442,070	299	9305	57	460,107	157	10,012
10	442,387	382	8890	28	460,285	201	9794
9	442,787	382	8890	27	460,505	201	9794
8	443,186	587	7868	26	460,725	201	9794
7	443,793	643	7585	25	460,945	407	8764
6	444,457	643	7585	24	461,370	407	8764
5	445,121	643	7585	23	461,795	951	6054
4	445,785	643	7585	22	462,763	951	6054
3	446,449	643	7585	51	463,732	951	6054
2	447,113	643	7585	50	464,869	643	7585
1	447,777	154	10,027	49	465,533	643	7585
73	448,211	215	9721	48	466,197	845	6582
72	448,446	215	9721	47	467,062	845	6582
71	448,681	215	9721	46	468,197	845	6582
70	448,916	485	8375	45	469,062	845	6582
69	449,420	564	7983	44	469,927	845	6582
68	450,002	564	7983	43	470,792	1199	4818
67	450,584	177	9912	42	472,012	1199	4818
66	450,780	243	9584	41	473,231	1199	4818
65	451,041	585	7877	40	474,451	1199	4818
64	451,644	664	7484	39	475,670	1199	4818
21	452,328	664	7484	38	476,890	2061	540
19	453,012	664	7484	37	478,972	2061	540
18	453,696	299	9305	36	481,053	2061	540
17	454,016	382	8890				

**Table 5:** The working strategies for minimum overall energy consumption routing in Layer A.

Node No.	Arrival Time (s)	Recharging Duration (s)	Remaining Battery Energy (J)	Node No.	Arrival Time (s)	Recharging Duration (s)	Remaining Battery Energy (J)
81	709,640	195	9820	22	733,149	340	9099
80	709,848	316	9217	21	733,504	340	9099
79	710,178	316	9217	20	733,856	340	9099
78	710,507	316	9217	19	734,208	340	9099
77	710,833	316	9217	18	734,560	340	9099
76	711,160	316	9217	17	734,913	340	9099
75	711,486	316	9217	16	735,265	340	9099
48	711,816	316	9217	92	735,818	340	9099
47	712,146	316	9217	91	736,171	340	9099
46	712,476	316	9217	90	736,523	340	9099
45	712,806	501	8296	89	736,876	495	8324
44	713,321	614	7729	88	737,384	630	7651
43	713,949	614	7729	87	738,027	630	7651
42	714,575	614	7729	86	738,669	630	7651
41	715,202	614	7729	85	739,312	630	7651
40	715,828	180	9898	84	739,956	108	10,259
15	716,168	315	9225	83	740,077	179	9904
14	716,498	315	9225	82	740,270	179	9904
13	716,827	315	9225	39	740,463	179	9904
12	717,156	315	9225	38	740,656	179	9904
11	717,485	503	8283	37	740,849	179	9904
10	718,003	595	7822	36	741,042	506	8269
9	718,612	595	7822	35	741,563	506	8269
8	719,222	595	7822	34	742,083	506	8269
7	719,832	595	7822	33	742,603	1122	5196
6	720,442	595	7822	32	743,740	1122	5196
5	721,052	595	7822	31	744,876	1122	5196
4	721,662	595	7822	74	746,178	1122	5196
3	722,272	595	7822	73	747,314	573	7936
2	722,882	595	7822	72	747,901	573	7936
1	723,492	161	9993	71	748,487	573	7936
106	723,927	260	9496	70	749,074	714	7231
105	724,201	260	9496	69	749,802	714	7231
104	724,476	260	9496	68	750,798	714	7231
103	724,751	260	9496	67	751,524	714	7231
102	725,025	468	8460	66	752,251	714	7231
101	725,507	595	7822	65	752,977	714	7231
100	726,117	595	7822	64	753,703	714	7231
99	726,725	595	7822	63	754,431	714	7231
98	727,334	142	10,088	62	755,159	1279	4414
97	727,489	248	9556	61	756,451	1279	4414
96	727,751	248	9556	60	757,744	1279	4414
95	728,012	463	8484	59	759,037	1279	4414
94	728,489	591	7847	58	760,330	1279	4414
93	729,092	591	7847	57	761,622	1279	4414
30	729,697	591	7847	56	762,915	1279	4414
29	730,302	591	7847	55	764,208	2057	540
28	730,907	591	7847	54	766,279	2057	540
27	731,513	205	9772	53	768,350	2057	540
26	731,732	340	9099	52	770,422	2057	540
25	732,086	340	9099	51	772,493	2057	540
24	732,441	340	9099	50	774,565	2057	540
23	732,795	340	9099	49	776,636	2057	540

The optimal traveling path for the recharging locomotive is calculated according to Theorem 4 and is shown in Figure 6. The length of this path is 13,980 m, and the time spent on traveling around it is 2796 s.

The orders of nodes listed in these two tables are according to their visiting orders along the traveling path, that is the first node along the roaming path of the recharging locomotive in Table 4 is the node with number 56, and the second one is the node with number 55. The second column stands for the arrival time of the recharging locomotive in the second working cycle. The third column tells us how long this node should be recharged. The forth column shows the remaining energy of each node in the second working cycle.

The lengths of each working cycle for the minimum hops and minimum overall energy consumption routing are about 4.3 × 10^5^ and 7.1 × 10^5^ s. The $\xi_{S}$ for both of them is 88.52% and 90.22%, which means the minimum overall energy consumption recharging scheme achieves better $\xi_{S}$ than the minimum hop routing recharging scheme.

**Figure 7:**
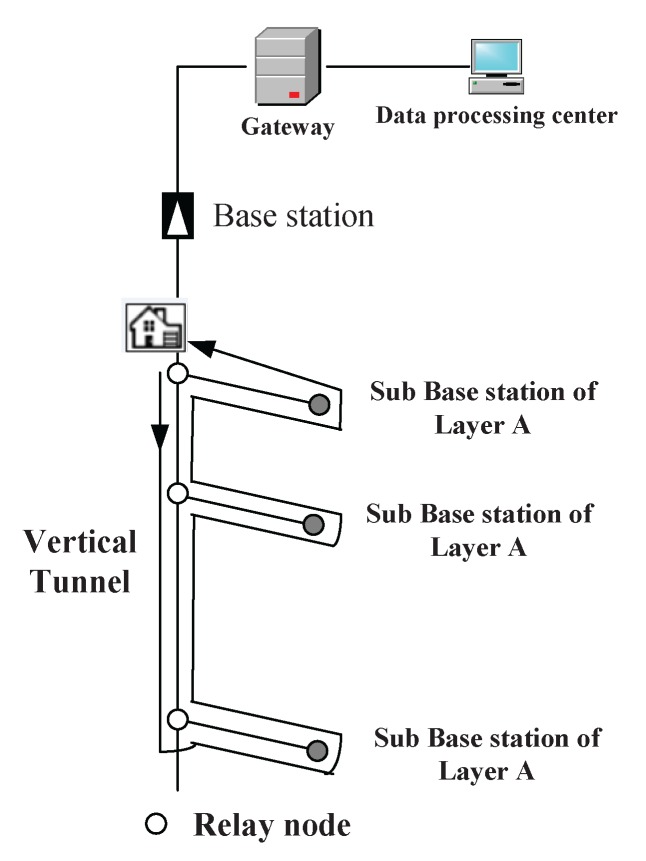
The optimal traveling path for the recharging locomotive in the vertical tunnel.

For the nodes in the vertical tunnel, we also apply the same approach. The optimal traveling path is shown in Figure 7. The corresponding working strategies after solving OPT-9 are shown in Table 6 and Table 7. The $\xi_{S}$ for both of them is 72.93% and 77.23%, which indicates that the minimum energy consumption recharging scheme also yields a better objective value.

**Table 6:** The working strategies for minimum hop routing in the vertical tunnel.

Node No.	Arrival Time (s)	Recharging Duration (s)	Remaining Battery Energy (J)	Node No.	Arrival Time (s)	Recharging Duration (s)	Remaining Battery Energy (J)
5	127,060	814	6755	27	142,696	972	5975
4	127,894	1040	5641	26	143,687	972	5975
3	128,954	1040	5641	25	144,678	972	5975
2	130,015	1040	5641	24	145,669	993	5872
1	131,075	1040	5641	23	146,681	993	5872
31	132,235	745	7094	22	147,693	993	5872
30	133,001	952	6072	21	148,705	993	5872
29	133,973	952	6072	20	149,717	993	5872
12	134,946	952	6072	19	150,729	993	5872
11	135,918	952	6072	18	151,741	993	5872
10	136,891	952	6072	17	152,753	993	5872
9	137,863	952	6072	16	153,765	993	5872
8	138,836	725	7191	15	154,776	2086	540
7	139,582	972	5975	14	156,881	2086	540
6	140,574	972	5975	13	158,986	2086	540
28	141,705	972	5975				

**Table 7:** The working strategies for minimum overall energy consumption routing in the vertical tunnel.

Node No.	Arrival Time (s)	Recharging Duration (s)	Remaining Battery Energy (J)	Node No.	Arrival Time (s)	Recharging Duration (s)	Remaining Battery Energy (J)
8	205,719	511	8249	37	225,430	1076	5443
7	206,243	877	6430	36	226,523	1076	5443
6	207,133	877	6430	35	227,615	1076	5443
5	208,023	877	6430	34	228,708	1076	5443
4	208,914	877	6430	33	229,800	1076	5443
3	209,804	877	6430	32	230,892	1162	5018
2	210,694	877	6430	31	232,071	1162	5018
1	211,584	877	6430	30	233,249	1162	5018
41	212,576	657	7520	29	234,427	1162	5018
40	213,249	993	5857	28	235,605	1162	5018
39	214,258	993	5857	27	236,784	1162	5018
38	215,266	993	5857	26	237,961	1162	5018
17	216,275	993	5857	25	239,138	1162	5018
16	217,284	993	5857	24	240,316	1162	5018
15	218,293	993	5857	23	241,493	1162	5018
14	219,302	993	5857	22	242,670	1162	5018
13	220,310	993	5857	21	243,847	2072	540
12	221,319	677	7421	20	245,935	2072	540
11	222,013	1076	5443	19	248,022	2072	540
10	223,105	1076	5443	18	250,109	2072	540
9	224,198	1076	544				

**Figure 8:**
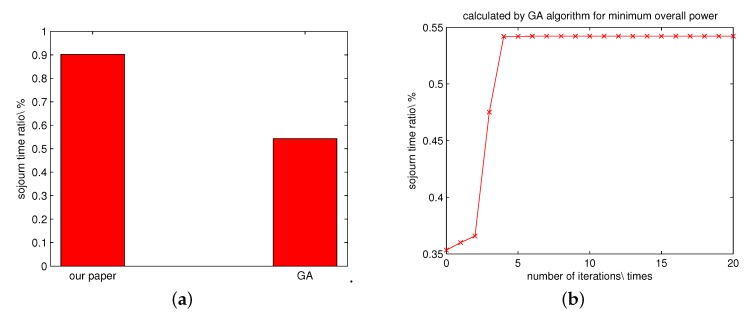
The caparison of the results obtained by solving the linear programming problem and the GA. (**a**) The comparison of the objective values obtained by solving the linear programming problem and the GA. (**b**) The convergence of the GA.

We also compare our solution via solving the linear programming problem with the one solved by the genetic algorithm. The optimal value figured out by the GA is around 55%. The comparison and the convergence of the GA are shown in Figure 8.

**Figure 9:**
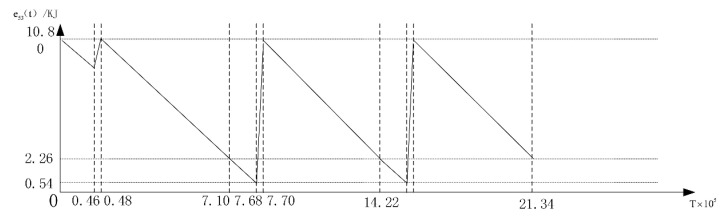
The energy consumption time curve of the node with the number 53 in Layer A.

Up to now, we have almost finished our discussion on working strategies for wireless rechargeable sensor networks in coal mines. However, this first working cycle is a little bit different from the rest, since at the very beginning of the first cycle, the energy of each node is $E_{max}$, which is higher than that at the beginning of the rest cycles. However, if we adjust the recharging power according to:

(22) $$\begin{array}{c} U_{i}^{1st}=U-\frac{E_{max}-E_{i}}{\tau_{i}} \end{array}$$

where $E_{i}$ stands for the beginning energy of node *i* at the rest cycles, then we can make the first and second cycle match perfectly. The energy consumption-time chart for the node with the number 53 is drawn Figure 9, and the recharging power for this nodes is adjusted to 0.76 Watts. From this figure, we can see that the initial energy level of this sensor node in the first working cycle is higher that that of the rest cycles.

## 7. Conclusions

Recharging sensor nodes deployed in coal mines remotely by implementing the wireless energy transfer technique may provide a promising way to make a wireless sensor network work perpetually. In this paper, we discuss problems on the relay nodes’ placement and the working strategies for wireless rechargeable sensor networks in coal mines in order to keep nodes from malfunctioning caused by insufficient energy supply. Two sets of optimization problems are introduced to obtain the optimal solutions to these two problems. For the problems related to the best relay nodes’ placement, we resort to the Lagrange dual problem and KKT conditions for help. The optimization problems formulated for the optimal working strategies for wireless rechargeable sensor networks are simplified and linearized through several properties and theorems. In the simulation section, the optimal solutions to these problems are provided and compared to the optimal solutions with respect to minimum hop routing schemes.

## Appendix A. The Strong Duality

**Theorem** **A1.** *The strong duality of the prime optimization problem OPT-2 and its Lagrange dual problem holds, i.e., the optimal values of these two problems are identical*.
**** The proof of this theorem is a little bit complicated. Before we move forward, we firstly rewrite OPT-2 as follows:

$$\begin{array}{c} \begin{array}{ll} \text{min} & g(\vec{d})\\ s.t. & A^{T}\vec{d}-L=0\\ & g_{i}\left(\vec{d}\right)\leq 0,i=0,\cdots ,2n+1 \end{array} \end{array}$$

where $\vec{d}$ is a column vector, which is ${\left\{d_{0},d_{1},\cdots ,d_{n}\right\}}^{T}$, and A is a column vector of which the entries are all one. The objective function $g(\vec{d})$ stands for $\Phi_{2}f\sum_{i=0}^{n}d_{i}^{k}$. For 0≤i≤n, the inequality constraints stand for $d_{i}-R\leq 0$. For n+1≤i≤2n+1, $g_{i}\left(\vec{d}\right)$ stands for $-d_{i-(n+1)}\leq 0$.

First of all, we denote the feasible solution set to OPT-2 as D. Then, we have the following facts:

- It is obvious that D is not empty, that is it has interior points. Additionally, we have certain $\vec{d}\in \text{int}D$, such that $g_{i}(\vec{d})<0$ and $A^{T}\vec{d}=L$ (intD stands for the set of interior points of D).
- The function $g(\vec{d})$ and $g_{i}\left(\vec{d}\right)$ are all convex functions for $\vec{d}\in \text{int}D$, and the rank of A is one.

We denote the optimal value of OPT-2 as $p^{★}$, and we denote the set A as:

(A1) $$\begin{array}{c} A=\left\{g_{0}\left(\vec{d}\right),g_{1}\left(\vec{d}\right),\cdots ,g_{2n+1}\left(\vec{d}\right),A^{T}\vec{d},g\left(\vec{d}\right))\right\} \end{array}$$

Then, we have:

(A2) $$\begin{array}{c} p^{★}=\text{inf}\left\{t|(u,w,t)\in A,u\leq 0,w=0\right\} \end{array}$$

where $u=\left\{g_{0}\left(\vec{d}\right),g_{1}\left(\vec{d}\right),\cdots ,g_{2n+1}\left(\vec{d}\right)\right\}$, $w=A^{T}\vec{d}$ and $t=g(\vec{d})$. The Lagrangian dual function of OPT-2 can be written as:

(A3) $$\begin{array}{c} G\left(\lambda ,v\right)=G\left(\lambda_{1,i},\lambda_{2,i},v\right)=\text{inf}\left\{{\left(\lambda ,v,1\right)}^{T}\left(u,w,1\right)|\left(u,w,t\right)\in A\right\} \end{array}$$

Now, we define the “epigraph” (the word “epigraph” means “upper graph”) of set A as:

(A4) $$\begin{array}{c} A_{E}=A+\left(R_{+}^{2n+2}\times \left\{0\right\}\times R_{+}\right) \end{array}$$

The convexity of the set $A_{E}$ can be justified easily through the definition of convex sets. We define another convex set:

(A5) $$\begin{array}{c} B=\left\{\left(\vec{0},0,s\right)\in R^{2n+2}\times R\times R|s<p^{★}\right\} \end{array}$$

Then, we can prove that the two convex sets, $A_{E}$ and B, do not intersect.

Suppose that $A_{E}\cap B\neq \emptyset$; then, we have at least one element $\left(u,w,t\right)\in A_{E}\cap B$. Therefore, we have u=0,w=0 and $t<p^{★}$. Since $\left(u,w,t\right)\in A_{E}$, then we have at least one $\vec{d}$, such that $g_{i}\left(\vec{d}\right)\leq 0$, $A^{T}\vec{d}=0$ and $g\left(\vec{d}\right)\leq t<p^{★}$, which is impossible, since $p^{★}$ is already the optimal solution to OPT-2. Hence, $A_{E}\cap B=\emptyset$. Since $A_{E}$ and B are both convex sets that do not intersect, there must exist a separating hyperplane between them. Therefore, there exists a non-zero vector $\left(\tilde{\vec{\lambda }},\tilde{v},\mu \right)$ and a non-negative number *α*, such that:

(A6) $$\begin{array}{c} \forall \left(u,w,t\right)\in A_{E}\Rightarrow {\left(\tilde{\vec{\lambda }},\tilde{v},\mu \right)}^{T}\left(u,w,t\right)\geq \alpha \end{array}$$

(A7) $$\begin{array}{c} \forall \left(u,w,t\right)\in B\Rightarrow {\left(\tilde{\vec{\lambda }},\tilde{v},\mu \right)}^{T}\left(u,w,t\right)\leq \alpha \end{array}$$

From Equation (A6), we have $\tilde{\vec{\lambda }}⪰0$ and μ≥0, otherwise ${\left(\tilde{\vec{\lambda }},\tilde{v},\mu \right)}^{T}\left(u,w,t\right)$ is unbounded below. From Equation (A7), we have μt≤α for any $t<p^{★}$. Therefore, we have $\mu p^{★}\leq \alpha$ since if $\mu p^{★}>\alpha$, then there must exist a vicinity of $p^{★}$, denoted as $\delta (p^{★})$, such that for any $x\in \delta (p^{★})$, μx>α, which contradicts that μt≤α for any $t<p^{★}$. Therefore, we have:

(A8) $$\begin{array}{c} \sum_{i=0}^{2n+1}{\tilde{\lambda }}_{i}g_{i}\left(\vec{d}\right)+\tilde{v}\left(A^{T}\vec{d}-L\right)+\mu g_{i}\left(\vec{d}\right)\geq \alpha \geq \mu p^{★} \end{array}$$

- If μ>0, for all $\vec{d}\in D$
  (A9) $$\begin{array}{c} \sum_{i=0}^{2n+1}\frac{{\tilde{\lambda }}_{i}}{\mu }g_{i}\left(\vec{d}\right)+\frac{\tilde{v}}{\mu }\left(A^{T}\vec{d}-L\right)+g_{i}\left(\vec{d}\right)=L\left(\vec{d},\frac{\tilde{\vec{\lambda }}}{\mu },\frac{v}{\mu }\right)\geq p^{★} \end{array}$$
  Let $\frac{{\tilde{\lambda }}_{i}}{\mu }=\lambda$ and $\frac{v}{\mu }=v$; we have $G\left(\lambda ,v\right)={\text{inf}}_{d}\left\{L(\vec{d},\tilde{\vec{\lambda }},v)\right\}$. Thus, we have $G\left(\lambda ,v\right)\geq p^{★}$. However, G(λ,v) always provides a lower bound of OPT-2; we have $G\left(\lambda ,v\right)\leq p^{★}$. Therefore, $G\left(\lambda ,v\right)=p^{★}$.
- If μ=0, from Equation (A8), we have:
  (A10) $$\begin{array}{c} \sum_{i=0}^{2n+1}{\tilde{\lambda }}_{i}g_{i}\left(\vec{d}\right)+\tilde{v}\left(A^{T}\vec{d}-L\right)\geq \alpha \geq 0 \end{array}$$
  By applying this to the point $\vec{d}$ that satisfies the first fact we mentioned above, we have:
  (A11) $$\begin{array}{c} \sum_{i=0}^{2n+1}{\tilde{\lambda }}_{i}g_{i}\left(\vec{d}\right)\geq 0 \end{array}$$
  Since $\tilde{\vec{\lambda }}⪰0$ and $g_{i}\left(\vec{d}\right)<0$, we must have $\tilde{\vec{\lambda }}=\vec{0}$. Since $\left(\tilde{\vec{\lambda }},\tilde{v},\mu \right)$ is a non-zero vector, we have $\tilde{v}\neq 0$. Since $\tilde{\vec{\lambda }}=\vec{0}$, Equation (A10) can be rewritten as:
  (A12) $$\begin{array}{c} \tilde{v}\left(A^{T}\vec{d}-L\right)\geq 0 \end{array}$$
  For those $\hat{\vec{d}}$ that satisfy the first fact, we have:
  (A13) $$\begin{array}{c} \tilde{v}\left(A^{T}\hat{\vec{d}}-L\right)=0 \end{array}$$
  Since $\tilde{v}\left(A^{T}\vec{d}-L\right)$ is an affine function of $\vec{d}$, and $\vec{d}\in \text{int}D$, there must exist some ${\vec{d}}^{\prime }$ within the vicinity of $\hat{\vec{d}}$, such that $\tilde{v}\left(A^{T}{\vec{d}}^{\prime }-L\right)<0$ unless $A\tilde{v}=0$. However, by the definition of A, which is a non-zero column vector, $A\tilde{v}=0$ holds only if $\tilde{v}=0$, which contradicts that $\left(\tilde{\vec{\lambda }},\tilde{v},\mu \right)$ is a non-zero vector. Therefore, *μ* cannot be zero.

In conclusion, we have $G\left(\vec{\lambda },v\right)=p^{★}$, which means that the strong duality holds. ☐

## Appendix B. The linearization Process of OPT-8

Let:

$$\left\{\begin{array}{ll} & \tau_{i}/\tau =\xi_{i},1\leq i\leq N\\ & \left(\tau_{S}+\tau_{P}\right)/\tau =\tau_{0}/\tau =\xi_{0}\\ & \tau_{S}/\tau =\xi_{S} \end{array}\right.$$

Then, the objective function of OPT-8 can be rewritten as $\xi_{S}$, and the third constraint can be rewritten as $\sum_{k=0}^{N}\xi_{k}=1$.

For the second constraint, we have:

(B1) $$\begin{array}{c} U\tau_{i}-\left(\rho r_{R}^{i}+\left(\Phi_{1}+\Phi_{2}d_{i}^{k}\right)r_{T}^{i}\right)\tau_{i}=\left(\rho r_{R}^{i}+\left(\Phi_{1}+\Phi_{2}d_{i}^{k}\right)r_{T}^{i}\right)\left(\tau -\tau_{i}\right)\leq E_{max}-E_{min} \end{array}$$

The “=” holds due to Equation (14). Then, by dividing the both sides with *τ*, we have:

(B2) $$\begin{array}{c} \xi_{0}-\xi_{S}-\left(\rho r_{R}^{i}+\left(\Phi_{1}+\Phi_{2}d_{i}^{k}\right)r_{T}^{i}\right)\left(1-\xi_{i}\right)\frac{\tau_{P}}{E_{max}-E_{min}}\geq 0 \end{array}$$

Thus, we have the linear programming problem OPT-9:

$$\begin{array}{c} \begin{array}{ll} \text{max} & \xi_{S}\\ s.t. & r^{i}+r_{R}^{i}=r_{T}^{i}\\ & \xi_{0}-\xi_{S}-\left(\rho r_{R}^{i}+\left(\Phi_{1}+\Phi_{2}d_{i}^{k}\right)r_{T}^{i}\right)\left(1-\xi_{i}\right)\frac{\tau_{P}}{E_{max}-E_{min}}\geq 0\\ & \sum_{k=0}^{N}\xi_{k}=1\;. \end{array} \end{array}$$
